# Intermittent theta burst stimulation enhances the efficacy of brain–computer interface in upper limb rehabilitation post-stroke

**DOI:** 10.3389/fneur.2026.1839697

**Published:** 2026-06-19

**Authors:** Xiaoqian Xia, Xiaoyu Kang, Lingyun Jia, Qianhui Wang, Linyao Zhang, Ruoqing Zhang, Yizheng Wang, Xiaoli Wu, Xiaogang Chen, Lixu Liu

**Affiliations:** 1 School of Rehabilitation Medicine, Capital Medical University, Beijing, China; 2 Department of Physical Therapy, Beijing Bo’ai Hospital, China Rehabilitation Research Center, Beijing, China; 3 School of Rehabilitation Medicine, The Second Affiliated Hospital of Wenzhou Medical University, Wenzhou, China; 4 Institute of Biomedical Engineering, Chinese Academy of Medical Sciences and Peking Union Medical College, Tianjin, China; 5 Neurorehabilitation Department, Beijing Bo’ai Hospital, China Rehabilitation Research Center, Beijing, China

**Keywords:** brain–computer interface, event-related desynchronization, intermittent theta burst stimulation, neuroplasticity, stroke rehabilitation, upper limb function

## Abstract

**Background:**

“BCI illiteracy,” characterized by insufficient *μ*-rhythm Event-Related Desynchronization (ERD) in approximately 40% of stroke patients, limits the efficacy of Brain–Computer Interface (BCI) training. Intermittent Theta Burst Stimulation (iTBS) can modulate cortical excitability. We hypothesized that sequential application of iTBS over the affected primary motor cortex (M1) before BCI training may enhance cortical activation, improve BCI decoding efficiency, and thereby promote upper limb motor recovery after stroke.

**Methods:**

This exploratory single-center randomized controlled trial (RCT) enrolled 18 subacute stroke patients, randomized to: BCI group (conventional rehab + BCI training) or iTBS + BCI group (conventional rehab + iTBS applied to the affected M1 cortex followed sequentially by BCI training). Interventions occurred 10 times over 2 weeks. Primary outcome: Fugl-Meyer Assessment - Upper Extremity (FMA-UE) score. Secondary outcomes: Modified Barthel Index (MBI), BCI task accuracy (BCI-TA). Mechanistic measures: sensorimotor cortex ERD and Laterality Index (LI).

**Results:**

In this exploratory study, FMA-UE improvement was greater in the iTBS + BCI group, with significant differences at week 4 (*Z* = 2.569, *p* = 0.008). iTBS + BCI group showed a greater BCI-TA increase (87.22 ± 10.83% vs. 68.24 ± 5.75%, *p* = 0.041), which correlated negatively with attention improvement (Schulte test time reduction; *r* = −0.796, *p* < 0.001). Only the iTBS+BCI group demonstrated deeper ERD over the affected sensorimotor cortex (C4; *p* = 0.001) and a shift in LI towards the affected side (*p* = 0.017) during affected hand motor imagery.

**Conclusion:**

This exploratory study suggests that sequential iTBS combined with BCI may have potential benefits in enhancing upper limb function in stroke patients. It boosts affected cortical excitability, improves BCI decoding efficiency, and remodels motor network activation, offering a new strategy to overcome “BCI illiteracy.”

**Clinical trial registration:**

https://www.chictr.org.cn/showproj.html?proj=173657, Identifier: ChiCTR2300069203.

## Introduction

According to the latest data from the Global Burden of Disease Study (GBD) 2021, stroke ranks as the third leading cause of death globally and the primary cause of disability. In 2021, there were 11.9 million new stroke cases globally, 7.3 million deaths (accounting for 10.7% of all-cause mortality), resulting in 160.5 million Disability-Adjusted Life Years (DALYs) (1). Approximately 70% of stroke survivors experience upper limb motor dysfunction, severely impacting their ability to perform Activities of Daily Living (ADL) such as eating, grooming, and dressing, thereby imposing a burden on families and society (2). Consequently, the recovery of upper limb motor function has become one of the core goals of stroke rehabilitation. Although traditional rehabilitation techniques such as Task-Oriented Training (TOT), Functional Electrical Stimulation (FES), and Electromyographic Biofeedback (EMG-BF) are widely used for upper limb functional recovery post-stroke, their ability to promote corticospinal tract reorganization and synaptic plasticity is limited (3). This limitation often results in functional recovery primarily focused on proximal gross movements (e.g., shoulder flexion, elbow flexion/extension), with suboptimal improvements in distal fine motor skills (e.g., finger opposition, lateral pinch) and complex functional tasks (e.g., buttoning, writing), failing to meet patients’ functional needs for reintegration into social life (4).

Brain–Computer Interface (BCI), a groundbreaking technology at the intersection of artificial intelligence and rehabilitation medicine, has emerged as a potential neuromodulatory approach for improving motor functional recovery post-stroke (5–7). Hybrid BCI systems enhance motor control by acquiring and decoding electroencephalography (EEG) signals related to Motor Imagery (MI) and Steady-State Visual Evoked Potentials (SSVEP) in real-time, driving exoskeleton robots or FES to execute movements, while providing real-time feedback (8–10). This “central-peripheral-central” closed-loop training strengthens neural plasticity by reinforcing the interaction between brain and muscle signals, thereby promoting motor functional reconstruction (11). However, a significant proportion of BCI users (approximately 30% of healthy adults and 40% of stroke patients) cannot effectively control BCI systems (12). This phenomenon, termed “BCI illiteracy,” substantially limits the effectiveness of BCI training (13). Studies indicate that “BCI illiteracy” stems partly from cognitive decline and impaired attention in stroke patients hindering their ability to correctly understand and execute BCI commands, and primarily from insufficient Event-Related Desynchronization (ERD) of the EEG *μ* rhythm (14). The *μ* rhythm, typically observed in the sensorimotor cortex within the 8–13 Hz frequency band, attenuates during MI; this attenuation is termed ERD and reflects increased activation of the sensorimotor cortex. As the amplitude of μ rhythm ERD is crucial for BCI decoding accuracy, a lack of robust μ rhythm ERD can impede the BCI system’s ability to detect the user’s motor intention (15). The amplitude of μ rhythm ERD, associated with motor-related cortical activation, can be modulated using Non-Invasive Brain Stimulation (NIBS) technique (16). Therefore, previous research has proposed NIBS as a feasible method to enhance BCI performance and address “BCI illiteracy.” Transcranial Direct Current Stimulation (tDCS), a common form of NIBS, has been extensively studied for its effects on BCI training (17–19). Some studies have shown that anodal tDCS applied to the motor cortex can increase *μ* rhythm ERD and improve BCI accuracy in both healthy adults and stroke survivors (17, 18, 20). Unfortunately, this improvement in BCI accuracy has not consistently translated into enhanced functional recovery of upper limb motor function following BCI training post-stroke, potentially because anodal tDCS fails to promote BCI training-induced neuroplastic changes (17–19). Therefore, further investigation into the effects of other forms of NIBS on BCI training efficacy is warranted.

Repetitive Transcranial Magnetic Stimulation (rTMS) is another widely used NIBS technique that modulates the excitability balance between cerebral hemispheres and is often employed to regulate cortical activation states. Evidence from reviews and meta-analyses has demonstrated the efficacy of rTMS in neurological conditions, particularly in stroke patients (21, 22). Previous research has demonstrated that rTMS can enhance ERD of sensorimotor rhythms elicited by MI in BCI, improve BCI classification accuracy, increase motor cortex excitability and white matter integrity, and subsequently improve upper limb motor function and activities of daily living (23–26). However, this combined therapeutic approach currently faces several limitations: Firstly, existing studies generally have small sample sizes, limiting the robustness and generalizability of their findings; secondly, therapeutic efficacy remains controversial, with inconsistent conclusions across studies; thirdly, treatment effects are susceptible to stimulation parameters (e.g., frequency, intensity, number of pulses), and standardized protocols have not been established; additionally, the therapy duration is long, and patient tolerance is often suboptimal when BCI treatment is administered sequentially, potentially affecting treatment adherence and ultimate outcomes (23–25).

Theta burst stimulation (TBS), a modified form of rTMS, can induce longer-lasting neural effects with shorter durations and lower stimulation intensities compared to conventional rTMS protocols. When applied to the affected M1 cortex, intermittent TBS (iTBS) typically significantly increases cortical excitability for 20–30 min post-stimulation (27–29). Previous studies have demonstrated that iTBS applied to the affected M1 cortex not only improves motor function in stroke patients but also promotes cognitive recovery (30, 31). Theoretically, this sequential therapy might enhance ERD of sensorimotor rhythms elicited by MI in BCI, thereby improving BCI classification accuracy. However, this hypothesis currently lacks sufficient empirical support. Among existing studies, only one involved healthy subjects (*n* = 8), and it found no evidence that iTBS promotes acute neuroplastic changes (e.g., changes in oxyhemoglobin HbO₂ concentration or functional connectivity) following BCI training. Furthermore, findings derived from healthy populations are difficult to directly extrapolate to the stroke patient population (23).

Therefore, we hypothesize that sequential application of iTBS targeting the affected M1 cortex prior to BCI training will enhance *μ*-rhythm ERD in the affected sensorimotor cortex, thereby improving BCI decoding accuracy, overcoming “BCI illiteracy,” and ultimately achieving more significant improvements in patients’ upper limb motor function and activities of daily living (ADL) compared to BCI training alone. Given the paucity of empirical evidence supporting this sequential intervention in stroke populations, the present study is designed as an exploratory investigation to preliminarily validate the feasibility and potential efficacy of this combined iTBS-BCI strategy.

## Methods

### Participants

This single-center, randomized, controlled trial (RCT) was designed as an exploratory study, screening patients admitted to the Department of Neurological Rehabilitation, China Rehabilitation Research Center, from September 2023 to December 2024. The study was approved by the Medical Ethics Committee of China Rehabilitation Research Center (CRRC-IEC-RF-SC-005-01), registered with the Chinese Clinical Trial Registry (ChiCTR2300069203), and filed with the National Medical Research Registration and Filing Information System. The study adhered to the Declaration of Helsinki and Good Clinical Practice (GCP) guidelines. The sample size calculation was informed by past research studies that employed comparable endpoints (23, 32). All eligible participants or their legal guardians were fully informed of the research objectives, intervention measures, possible clinical benefits and potential risks, evaluation indicators, follow-up arrangements and personal privacy protection measures in detail. Written informed consent was obtained from all participants or their legal guardians before formal enrollment into the study.

Inclusion Criteria:(1) Met the World Health Organization (WHO) diagnostic criteria for stroke, first episode of unilateral ischemic or hemorrhagic stroke (confirmed by Computed Tomography/Magnetic Resonance Imaging [CT/MRI]); (2) In the subacute phase of stroke (1 week to 6 months post-onset) (33); (3) Aged between 18 and 70 years; (4) Brunnstrom stage for upper limb and hand ≤ Stage V, Modified Ashworth Scale (MAS) score ≤ Grade 2; (5) Mini-Mental State Examination (MMSE) score ≥ 22; (6) All participants or their legal guardians voluntarily signed written informed consent prior to study participation.

Exclusion Criteria: (1) Contraindications to rTMS (e.g., intracranial metal implants, history of epilepsy, etc.); (2) Comorbid other central/peripheral nervous system diseases or psychiatric disorders (schizophrenia, bipolar disorder, etc.); (3) Severe spasticity (MAS > Grade 3) or pain (Visual Analogue Scale [VAS] > 4 points) in the hemiplegic upper limb affecting motor function; (4) Non-stroke-related organic injury to the upper limb (e.g., fracture, tendon rupture); (5) Severe aphasia or comprehension impairment.

### Randomization and blinding

This study employed an assessor-blinded, randomized controlled design. A 1:1 random allocation sequence was computer-generated by an independent researcher not involved in participant recruitment or outcome assessment. Neither stratification nor block randomization was applied. Allocation concealment was achieved using the sequentially numbered, sealed opaque envelope method, group assignments were placed in sequentially numbered, opaque, sealed envelopes. A designated staff member opened the envelopes strictly in order of participant enrollment. Rehabilitation therapists were aware of group allocation but delivered interventions independently and were prohibited from disclosing any intervention-related information to participants. Blinding compliance was monitored by an independent staff member, and participants with broken blinding were excluded from analysis. All outcome assessors remained blinded to group assignment throughout the study, and all pre- and post-intervention assessments were performed by the same assessor to minimize intra-rater variability. Participants were randomly allocated into two groups. BCI Group (Group 1): Received conventional rehabilitation training combined with BCI training. iTBS + BCI Group (Group 2): Received conventional rehabilitation plus iTBS intervention applied to the affected M1 cortex, followed by BCI training within 30 min post-stimulation. All participants completed 10 intervention sessions (5 sessions per week for 2 weeks). The iTBS + BCI group strictly adhered to the sequential treatment model of “iTBS followed by BCI”.

### Intervention measures

#### BCI training

The study utilized a hybrid BCI system developed by the Institute of Biomedical Engineering, Chinese Academy of Medical Sciences, integrating dual-modality technology of MI and SSVEP. The system was equipped with 32-channel EEG electrodes arranged according to the international 10–20 system, sampled at 1000 Hz. The reference electrode was placed on the left mastoid and the ground electrode on the forehead.

**Figure 1:**
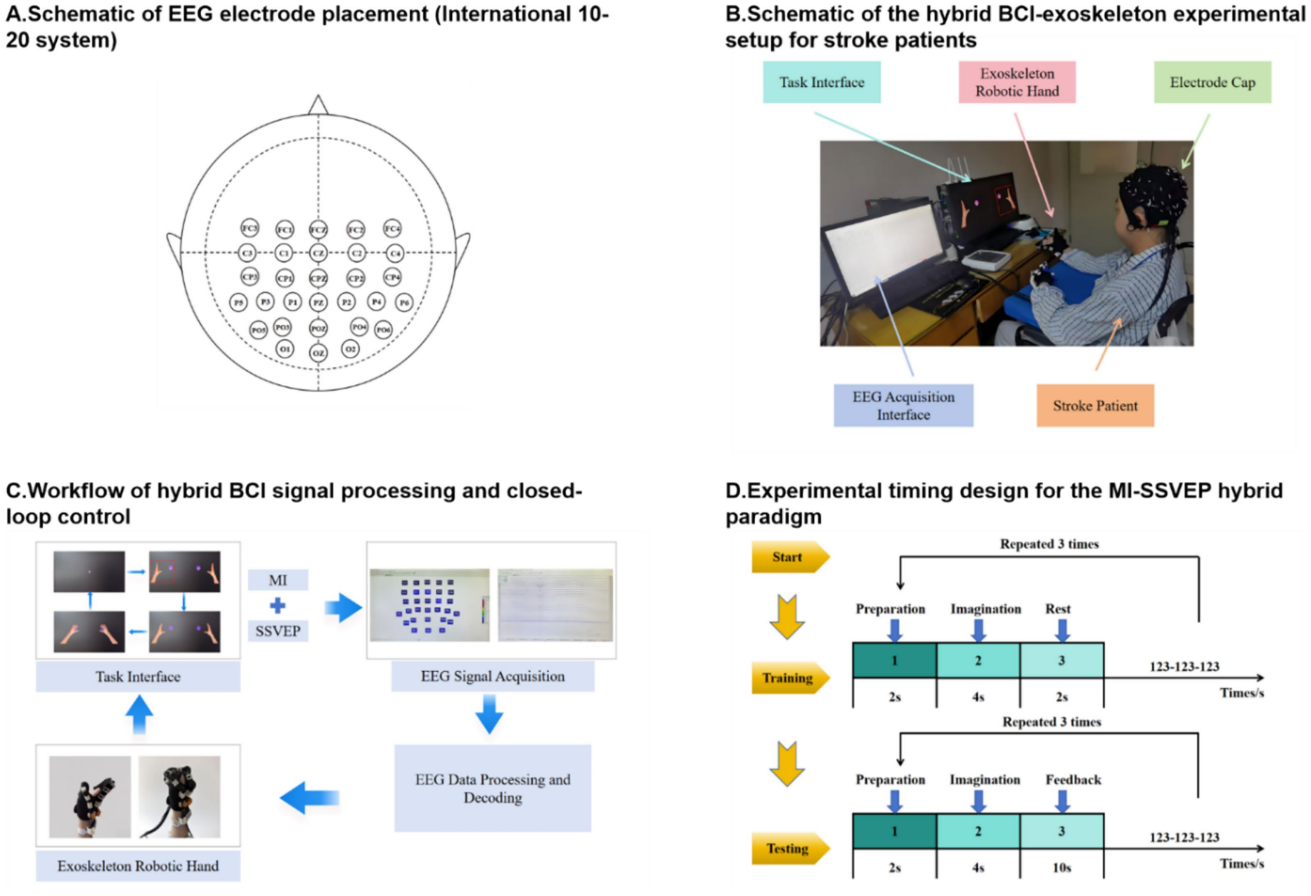
Experimental setup and workflow for hybrid BCI–exoskeleton system in stroke patients. **(A)** Schematic of EEG electrode placement (international 10–20 system). **(B)** Schematic of the hybrid BCI-exoskeleton experimental setup for stroke patients. **(C)** Workflow of hybrid BCI signal processing and closed–loop control. **(D)** Experimental timing design for the MI–SSVEP hybrid paradigm. BCI = Brain–Computer Interface; EEG = Electroencephalogram; MI = Motor Imagery; SSVEP = Steady - State Visual Evoked Potential.

The training protocol consisted of two phases: Calibration Phase: Patients performed hand MI (left/right hand grasping a ball) according to on-screen animated prompts. The system simultaneously analyzed EEG signals and established a classification threshold (accuracy threshold ≥50%). The experiment employed a structure of 3 blocks × 20 trials (left/right hand randomized alternation), with a single block duration of 3 min. Only MI was performed during this phase without exoskeleton robotic hand feedback. A 30-s rest period was provided after every 20 trials. Formal Training Phase: In a closed-loop control mode, patients directly triggered the exoskeleton robotic hand to execute corresponding actions by imagining left/right hand grasping a ball. The 3 blocks × 20 trials structure (left/right hand randomized alternation) was maintained. Correct imagination triggered feedback from the ipsilateral exoskeleton robotic hand (grasp/extend), while incorrect imagination triggered feedback from the contralateral hand. Single block duration was extended to 6 min. A 30-s rest interval was maintained after every 20 trials. After each imagination trial, the system output the patient’s MI accuracy. Total training duration per session was approximately 45 min (Figure 1).

#### iTBS

The QuiksVision Transcranial Magnetic Stimulation 3D Navigation System (Shenzhen Intelect Medical Technology Co., Ltd., China) ensured coil positioning accuracy (displacement <3 mm, angle deviation <3°). Stimulation was delivered using the M-100 Ultimate magnetic stimulator (Shenzhen Intelect Medical Technology Co., Ltd., China) with a figure-of-eight coil (diameter 90 mm) placed over the affected primary motor cortex (M1) at a 45° angle to induce posterior–anterior directed currents. Stimulation intensity started at 50% of the maximum stimulator output (MSO) and was increased in 10% increments. Electromyography (EMG) was recorded from target muscles (first dorsal interosseous and extensor carpi radialis). Intensity was gradually increased until a Motor Evoked Potential (MEP) was elicited or until 100% MSO was reached. MEP + was defined as: an MEP with peak-to-peak amplitude ≥50 μV and stable latency elicited in at least 5 consecutive stimulations (active or passive state) (33, 34).

**Figure 2:**
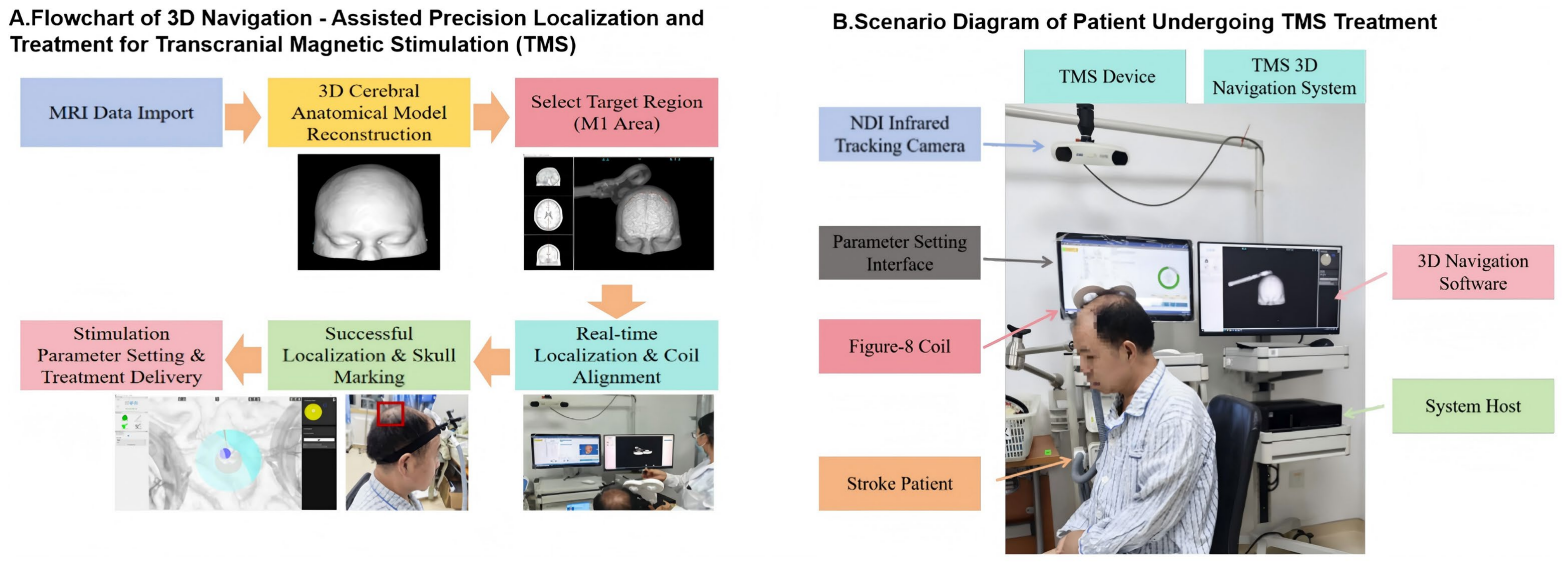
3D navigation–assisted transcranial magnetic stimulation (TMS) workflow and treatment scenario. **(A)** Flowchart of 3D navigation-assisted precision localization and treatment for TMS. The workflow begins with importing MRI data to reconstruct a 3D cerebral anatomical model. The target region (M1 area) is then selected, followed by setting stimulation parameters and delivering treatment. Key steps include successful localization with skull marking and real- time localization for coil alignment, ensuring precise TMS delivery. **(B)** Scenario diagram of patient undergoing TMS treatment. This diagram illustrates a stroke patient receiving TMS treatment. TMS = Transcranial Magnetic Stimulation; MRI = Magnetic Resonance Imaging.

Stimulation Parameters: 3 pulses per burst (50 Hz), burst interval 5 Hz (200 ms), 80% of Active Motor Threshold (AMT), total duration 190 s (600 pulses). Participants remained relaxed during stimulation (Figure 2) (34).

#### Conventional rehabilitation intervention

All participants received standardized conventional rehabilitation training during the intervention period, including basic physical therapy (e.g., range of motion training, muscle strength enhancement training) and occupational therapy (e.g., activities of daily living training). The training intensity and frequency were consistent across groups (30 min per session, 5 sessions per week for 2 weeks), ensuring balanced comparability in the dosage and core content of conventional rehabilitation intervention between the two groups to avoid interference with the primary outcome indicators of the study.

### Outcome measures

Primary outcome measure: Fugl-Meyer Assessment for Upper Extremity (FMA-UE): Quantified the degree of upper limb motor impairment, encompassing joint range of motion, coordination, and reflexes. The scoring range is 0–66 points, with higher scores indicating better upper limb motor function (35).

Secondary outcome measures: MAS: Assessed muscle tone level in the affected limb (36). Hamilton Anxiety Scale (HAMA): Assessed anxiety state (37). Hamilton Depression Scale (HAMD): Assessed depression state (37). Modified Barthel Index (MBI): Evaluated activities of daily living ability (38). Brain–Computer Interface Task Accuracy (BCI-TA): The percentage of valid trials where the system output correctly matched the patient’s motor imagery command of affected or unaffected hand grasp/extension in the online closed-loop feedback task. Schulte Grid Test: Assessed attention (39).

### Electroencephalogram data processing and analysis

(1) Data Acquisition and Preprocessing: EEG data from the calibration phase of the BCI system were collected for patients in the BCI group. Real-time EEG signals first underwent 0.5–30 Hz band-pass filtering and 50 Hz notch filtering to remove power line interference. Ocular and muscular artifacts were automatically removed using Independent Component Analysis (ICA). Preprocessed signals were segmented into 2-s epochs time-locked to the onset of motor imagery cues, with baseline correction performed using the 200 ms pre-cue period. A parallel decoding strategy was adopted for feature extraction and control command output: the Filter Bank Common Spatial Pattern (FBCSP) algorithm was used to extract motor imagery features (with 4 filter banks covering 8–30 Hz and 2 pairs of spatial filters), and the Filter Bank Canonical Correlation Analysis (FBCCA) algorithm was used to recognize Steady-State Visual Evoked Potential (SSVEP) signals (with 2 harmonics and a 1-s time window). Control commands were finally output via a weighted decision fusion module, with weights of 0.4 for motor imagery and 0.6 for SSVEP (9).

C3 and C4 channels were selected for subsequent analysis. Offline data processing was performed on the MATLAB (R2020a, MathWorks Inc., USA) platform. A fourth-order Butterworth bandpass filter was applied to extract three frequency bands: alpha (8–13 Hz), beta (13–30 Hz), and full alpha-beta (8–30 Hz). (2) Data Segmentation and Denoising: Filtered signals were squared, segmented, and epoched using a time window from 3 s before to 4 s after the cue ([−3,4] s), where time 0 corresponded to the MI command cue point. Trials with amplitudes exceeding ± 150 μV in the 0.1–30 Hz bandpass filtered signal were rejected as noise. The power time course in the 8–30 Hz band was manually reviewed for further denoising. (3) Feature Parameter Calculation: The power time course for each frequency band was averaged across trials and movement type (left/right). Event-Related Desynchronization/ Synchronization (ERD/ERS) values were calculated using Equation 1 as follows (40):

ERD/ERS=[(Pmov−Pref)/Pref]×100% (1)

Where Pmov is the median power within the [1,4] s time window, and Pref is the median power within the resting state [−3, −1] s time window. A negative ERD value indicates the percentage decrease in power during MI; a lower (more negative) value reflects greater cortical activation. For patients with right hemiplegia, ERD/ERS values from C3 and C4 channels and movement type labels were flipped to simulate left hemiplegia in all patients, simplifying result presentation and interpretation. The Laterality Index (LI) was calculated for each patient, trial, movement type, and frequency band using the following formula (40):

LI=(ERDi−ERDc)/(∣ERDi∣+∣ERDc∣) (2)

Where ERDi represents the ipsilesional ERD and ERDc represents the contralesional ERD. In this study context, since all patient data were transformed to simulate left limb paralysis (i.e., right hemisphere lesion), ERDi values were extracted from the C4 channel, and ERDc values were extracted from the C3 channel. According to Equation 2, an LI value approaching −1 indicates completely ipsilateral brain activation, while an LI value approaching 1 indicates completely contralateral activation.

### Statistical analysis

All statistical analyses in this study were performed based on the per-protocol (PP) dataset in accordance with the CONSORT guidelines. Participants who withdrew or dropped out after randomization were excluded from final statistical analysis. Normally distributed continuous data are presented as mean ± standard deviation (SD), and between-group comparisons were performed using independent samples t-tests. Non-normally distributed continuous data are presented as median and interquartile range (IQR), and between-group comparisons were performed using Mann–Whitney *U* tests. Categorical data are presented as number (%), and between-group comparisons were performed using χ^2^ tests. The Friedman test was used to analyze the within-group differences of outcome measures at three time points. The Mann–Whitney *U* test was applied to compare between-group differences at each time point with Bonferroni correction for multiple comparisons. Pearson correlation coefficient was used for correlation analysis. A two-sided *p*-value < 0.05 was considered statistically significant. Data analysis was performed using SPSS software (version 25.0). Figures were generated using GraphPad Prism software (version 10.3.0).

## Results

### Participant characteristics

**Figure 3:**
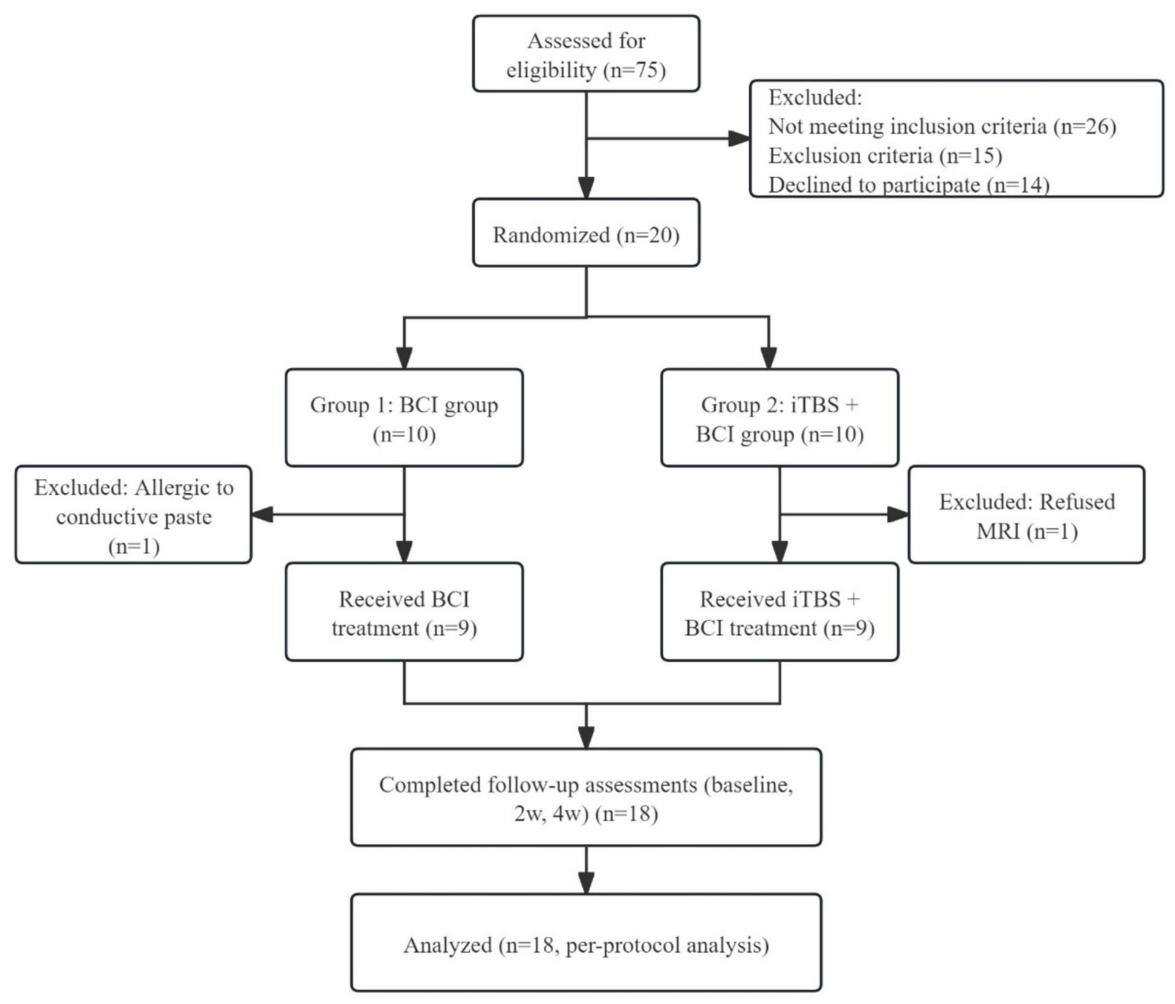
CONSORT flow diagram of participant recruitment and analysis. Initially, 75 individuals were assessed for eligibility. After excluding those not meeting inclusion criteria (*n* = 26), failing exclusion criteria (*n* = 15), or declining participation (*n* = 14), 20 eligible patients were randomized into two groups: BCI group (*n* = 10) and iTBS + BCI group (*n* = 10). During intervention, 1 participant in the BCI group was excluded due to allergy to conductive paste, and 1 in the iTBS + BCI group refused MRI, leaving 9 per group for treatment. Finally, 18 patients completed assessments at baseline, 2 weeks post-training, and 4 weeks follow - up. BCI = Brain–Computer Interface; iTBS = intermittent theta–burst stimulation.

A total of 18 patients were enrolled. Baseline demographic data and clinical scores showed no significant differences between the groups (Table 1). All participants underwent EEG examination; data from 2 participants were excluded due to technical issues, resulting in EEG data from 16 participants being included in the analysis (Figure 3). The treatment protocol was well tolerated, and no device-related malfunctions, safety concerns, or other adverse events were reported in either group.

**Table 1:** Characteristics of participants.

Demographic data	Subcategory	Group 1 (*n* = 9)	Group 2 (*n* = 9)	*p-*value
Age, y, mean (SD)		40.11 ± 17.95	38.44 ± 15.99	0.838
Level of education (%)	Primary school or below	0(0.00%)	2(22.2%)	0.961
	Secondary school	5(55.6%)	2(22.2%)	
	University	4(44.4%)	5(55.6%)	
Sex (%)	Male	7(77.8%)	6(66.7%)	1.000
	Female	2(22.2%)	3(33.3%)	
BMI, mean (SD)		22.04 ± 3.95	21.21 ± 3.88	0.661
Time from stroke to baseline assessment, d, median (IQR)		59.00(27.50, 113.50)	34.00(16.00, 93.50)	0.270
Type of stroke (%)	Ischemic	4(44.4%)	5(55.6%)	1.000
	Hemorrhagic	5(55.6%)	4(44.4%)	
Hypertension (%)	Yes	4(44.4%)	5(55.6%)	1.000
	No	5(55.6%)	4(44.4%)	
Type II diabetes (%)	Yes	2(22.2%)	3(33.3%)	1.000
	No	7(77.8%)	6(66.7%)	
Heart diseases (%)	Yes	0(0.00%)	1(11.1%)	1.000
	No	9(100%)	8(88.9%)	
Smoking history (%)	Yes	6(66.7%)	2(22.2%)	0.153
	No	3(33.3%)	7(77.8%)	
Side of lesion (%)	Left	5(55.6%)	3(33.3%)	0.637
	Right	4(44.4%)	6(66.7%)	
RMT, mean (SD)		45.78 ± 9.78	47.11 ± 9.52	0.773
NIHSS, median (IQR)		4.00(3.50, 5.00)	5.00(4.00, 6.00)	0.292
FMA-UE (SD)		20.89 ± 9.60	18.22 ± 9.54	0.563
MAS, median (IQR)		1.50(1.00, 2.00)	1.00(1.00, 1.25)	0.117
MBI, mean (SD)		68.44 ± 14.16	64.44 ± 11.58	0.521
HAMA, mean (SD)		7.33 ± 4.69	10.00 ± 7.86	0.395
HAMD, mean (SD)		10.67 ± 8.60	10.78 ± 8.23	0.978

BMI = Body Mass Index; NIHSS = National Institutes of Health Stroke Scale; FMA-UE = Fugl–Meyer Assessment of Upper Extremity; MAS = Modified Ashworth Scale; MBI = Modified Barthel Index; HAMA = Hamilton Anxiety Rating Scale; HAMD = Hamilton Depression Rating Scale; IQR = Interquartile Range; SD = Standard Deviation.

### FMA-UE

**Figure 4:**
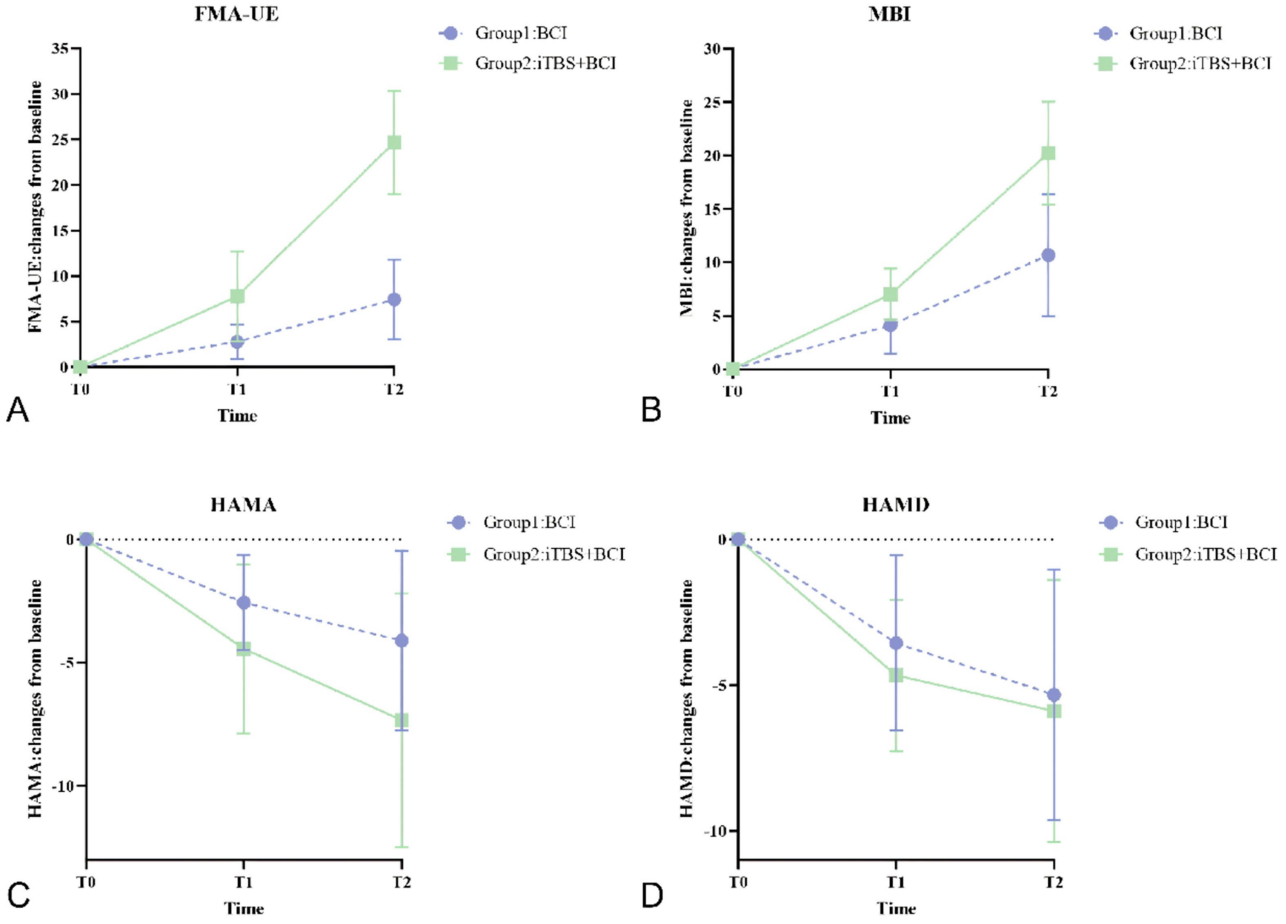
Temporal changes in outcome measures for BCI and iTBS + BCI groups. Plots depict the changes from baseline in four outcome measures across three time points (T0, T1, T2) for two groups: Group 1 (BCI alone) and Group 2 (iTBS + BCI). **(A–D)** Line graphs illustrate mean changes from baseline in **(A)** FMA-UE, **(B)** MBI, **(C)** HAMA, and **(D)** HAMD scores at baseline (T0), 2 weeks (T1), and 4 weeks (T2) post-randomization. Error bars represent standard deviation. BCI = Brain–Computer Interface; iTBS = intermittent theta-burst stimulation; FMA - UE = Fugl-Meyer Assessment of Upper Extremity; MBI = Modified Barthel Index; HAMA = Hamilton Anxiety Rating Scale; HAMD = Hamilton Depression Rating Scale.

Friedman tests revealed significant temporal changes in scores for both groups (all *p* < 0.001). Mann–Whitney U tests with Bonferroni correction indicated no intergroup difference at week 2 (*Z* = 0.443, *p* = 0.666). The iTBS+BCI group achieved notably higher FMA-UE scores at week 4 (*Z* = 2.569, *p* = 0.008) (Figures 4A,B).

The recognized MCID (minimal clinically important difference) of FMA-UE is about 5 points (41). The absolute improvement of 24.67 points in the iTBS + BCI group at week 4 obviously exceeded MCID, verifying that the functional gain has definite clinical reference significance.

**Figure 5:**
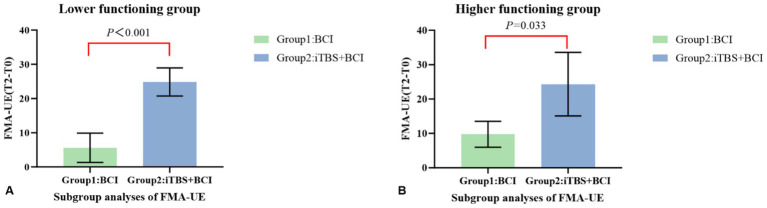
Subgroup analyses of Fugl-Meyer assessment of upper extremity (FMA–UE) Changes (T2 vs T0). Box plots illustrate the differences in FMA-UE scores (T2–T0) between Group 1 (BCI alone) and Group 2 (iTBS + BCI) across two functional subgroups. **(A)** Lower Functioning Group. The iTBS + BCI group shows a significantly larger improvement in FMA-UE scores compared to the BCI group. (*p* < 0.001). **(B)** Higher Functioning Group. The iTBS + BCI group also demonstrates greater FMA-UE score improvement than the BCI group, with a significant difference (*p* = 0.033), suggesting additional benefits of combining iTBS and BCI even in patients with relatively higher baseline functions. Error bars represent standard deviation. BCI = Brain–Computer Interface; iTBS = intermittent theta-burst stimulation; FMA–UE = Fugl–Meyer Assessment of Upper Extremity.

Patients were stratified by Brunnstrom stage into a lower-function group (Group 1: *n* = 4; Group 2: *n* = 3) and a higher-function group (Group 1: *n* = 5; Group 2: *n* = 6). Results showed that in both the lower-function and higher-function subgroups, the magnitude of FMA-UE score improvement at 4 weeks was significantly greater in Group 2 (iTBS + BCI) compared to Group 1 (BCI) (*P*_lower function_<0.001; *P*_higher function_ = 0.033) (Figures 5A,B). This suggests that regardless of baseline motor function level, the combination of iTBS and BCI therapy provided additional benefits for upper limb functional recovery.

### MBI

Friedman tests showed significant improvements over time in both groups (all *p* < 0.001). Mann–Whitney *U* tests showed no significant between-group differences at any time point (all *p* > 0.05).

### Other scale assessments (MAS, HAMA, HAMD)

Friedman tests revealed obvious temporal variations in MAS scores of both groups (*p* = 0.030 and *p* = 0.006). After Bonferroni correction, Mann–Whitney U test found no intergroup difference at week 2. The iTBS+BCI group had remarkably lower MAS scores at week 4 (*Z* = −2.437, *p* = 0.040).

HAMA and HAMD scores of the two groups declined markedly over time (all *p* < 0.05).

No statistically significant intergroup disparity was observed at any detection time (all *p* > 0.05) (Figures 4C,D).

### BCI accuracy and Schulte Grid Test

**Figure 6:**
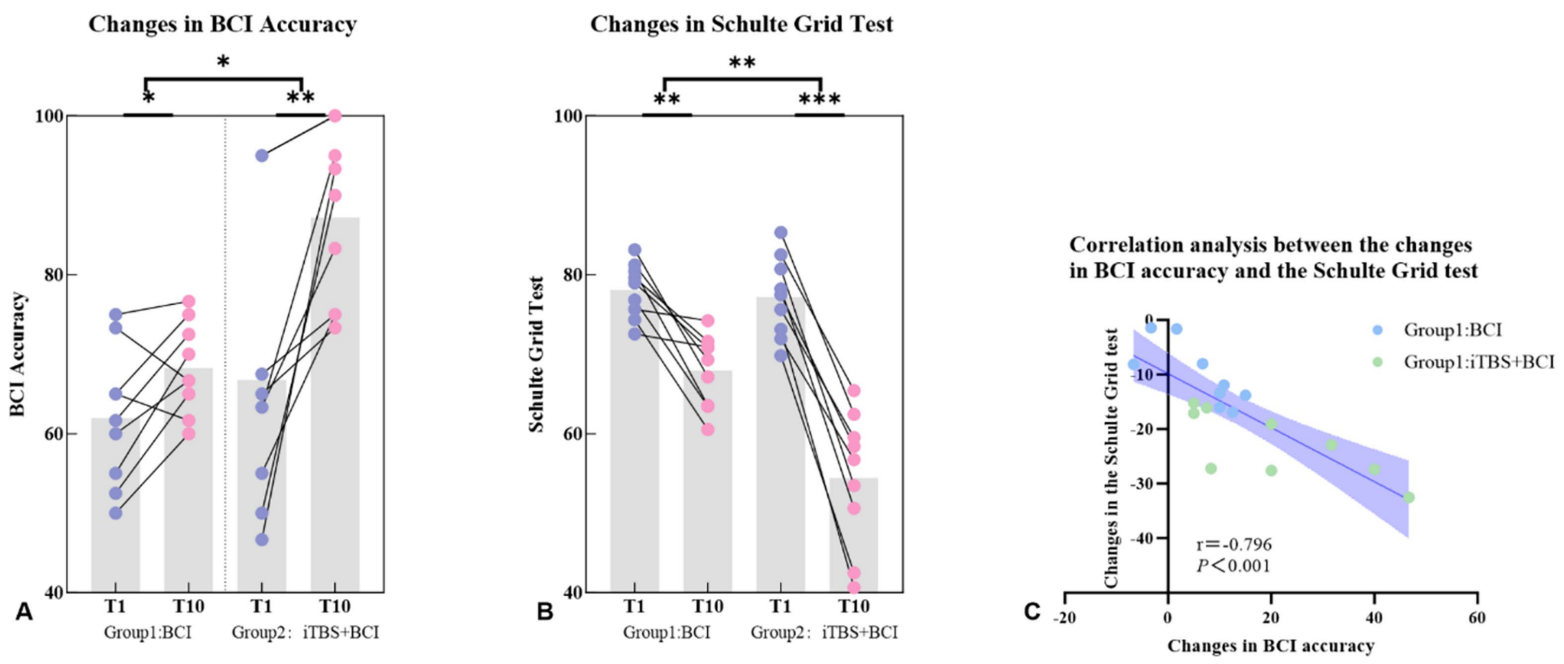
Changes in BCI performance and cognitive assessment, and their correlation. Plots illustrate changes in brain–computer interface (BCI) accuracy, results of the Schulte Grid Test and their correlation across two groups (Group 1: BCI alone; Group 2: iTBS + BCI). **(A)** Changes in BCI Accuracy. Connected dots represent individual participants’ BCI accuracy at T1 and T10. Group 2 (iTBS + BCI) shows a more pronounced increase in accuracy compared to Group 1 (BCI alone). **(B)** Changes in Schulte Grid Test. Individual data points (connected) depict performance changes on the Schulte Grid Test at T1 and T10. Group 2 demonstrates greater improvement in attention-related performance. **(C)** Correlation analysis between changes in BCI accuracy and Schulte grid test. A negative correlation is observed between changes in BCI accuracy and changes in the Schulte Grid Test (*r* = −0.796, *p* < 0.001), suggesting a relationship between enhanced BCI performance and improved attention (lower Schulte Grid Test scores indicate better attention). BCI = Brain–Computer Interface; iTBS = intermittent theta–burst stimulation; T1 = first training session; T10 = last training sesseion.*p < 0.05, **p < 0.01, ***p < 0.001.

BCI accuracy significantly improved after training in both groups. In Group 1, BCI accuracy was 61.94 ± 8.68% at T0 (baseline) and increased to 68.24 ± 5.75% at T10 (post 10 sessions), with a statistically significant within-group difference (*P₁* = 0.035). In Group 2, BCI accuracy was 66.76 ± 17.48% at T0 and reached 87.22 ± 10.83% at T10, also showing a significant within-group difference (*P₂* = 0.041). Comparison of the magnitude of improvement between groups showed that the increase in BCI accuracy was significantly greater in Group 2 than in Group 1 (*P₃* = 0.041) (Figure 6A).

The Schulte Grid Test also showed significant improvement. The test completion time in Group 1 decreased from 78.09 ± 3.48 s at T0 to 67.95 ± 4.57 s at T10 (*P₁* = 0.001). In Group 2, the completion time decreased from 77.20 ± 5.11 s at T0 to 54.41 ± 8.52 s at T10 (*P₂* < 0.001). Between-group comparison indicated that the improvement in Schulte Grid Test time (i.e., reduction in time) was significantly greater in Group 2 than in Group 1 (*P₃* = 0.002) (Figure 6B). Further analysis of the association between the change in BCI accuracy and the change in Schulte Grid Test time (Figure 6C) revealed a significant negative correlation (*r* = −0.796, *p* < 0.001). This indicates that a greater increase in BCI accuracy was associated with a greater reduction in Schulte Grid Test time (Figure 6C and Table 2).

**Table 2:** Changes in BCI accuracy and Schulte Grid Test results between T0 and T10.

Outcome measure	Group1			Group2			*P_3_*
	T0	T10	*P_1_*	T0	T10	*P_2_*	
BCI Accuracy	61.94 ± 8.68	68.24 ± 5.75	0.035	66.76 ± 17.48	87.22 ± 10.83	0.005	0.041
Schulte Grid Test	78.09 ± 3.48	67.95 ± 4.57	0.001	77.20 ± 5.11	54.41 ± 8.52	<0.001	0.002

P₁ and P₂ indicate intragroup differences (T10 vs T0) for Group 1 and Group 2, respectively. P₃ represents intergroup differences in changes from T0 to T10. BCI = Brain–Computer Interface; T0 = baseline; T10 = post-training.

### Motor-related sensorimotor ERD

**Figure 7:**
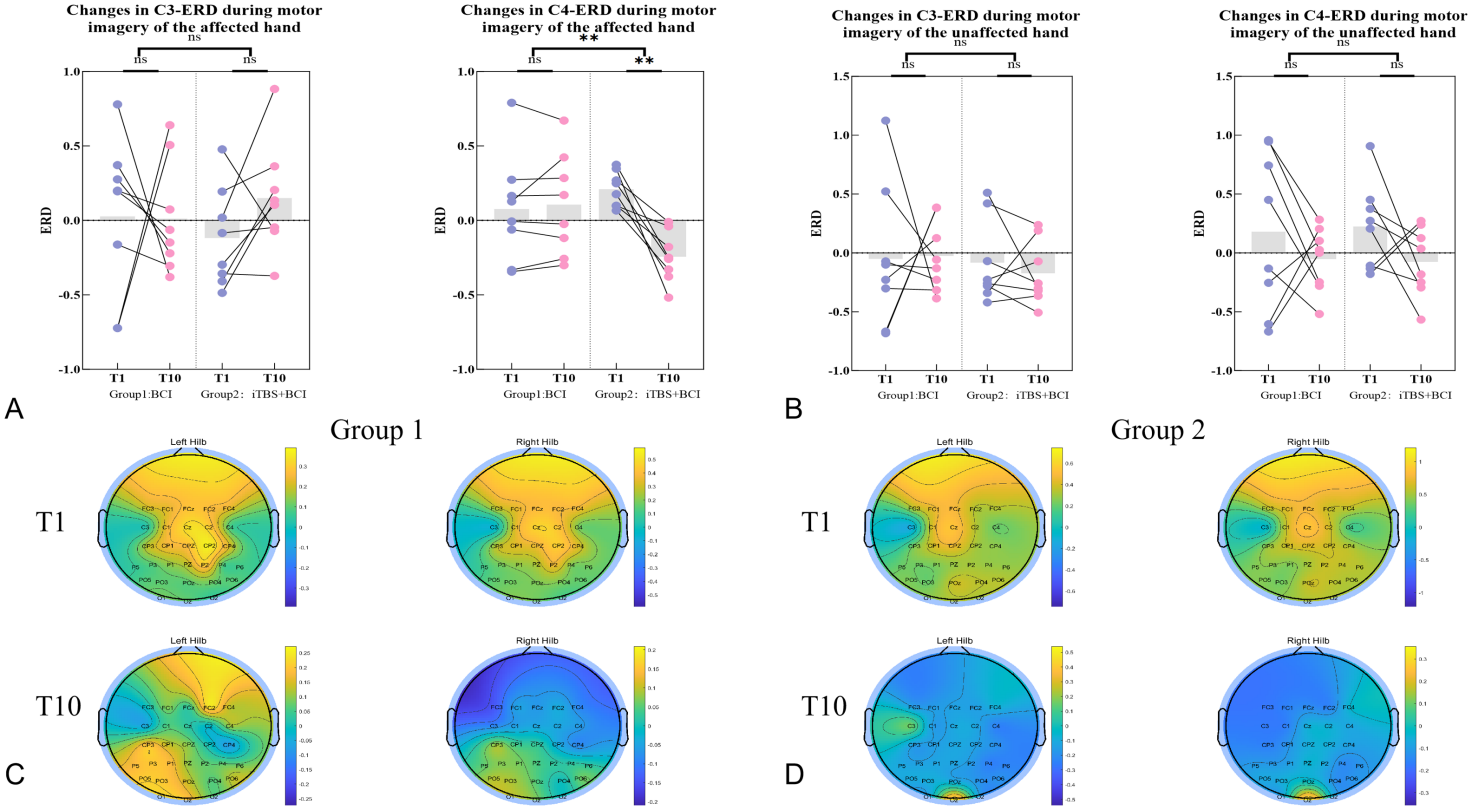
Changes in ERD during motor imagery and brain topographic maps. This figure presents the changes in ERD during motor imagery of the affected and unaffected hands, along with the brain topographic maps of electrical activity, for two groups (Group 1: BCI group; Group 2: iTBS + BCI group) at two points (T1, T10). **(A)** Changes in ERD at C3 and C4 electrodes during motor imagery of the affected hand. **(B)** Changes in ERD at C3 and C4 electrodes during motor imagery of the unaffected hand. ERD reflects the degree of cortical neural activation. Negative values indicate stronger desynchronization (increased neural activation), while positive values indicate synchronization (decreased activation). **(C)** Brain topographic maps of electrical activity for Group 1 (BCI Group) at T1 and T10. **(D)** Brain Topographic maps of electrical activity for Group 2 (iTBS + BCI Group) at T1 and T10. The maps depict the spatial distribution of brain electrical activity (ERD) for the respective groups at the initial (T1) and follow-up (T10) time points. Cool colors (blue) represent stronger ERD (suggesting higher neural activation during motor imagery), while warm colors (yellow/red) represent weaker ERD (lower neural activation). These maps illustrate the changes in neural activation patterns after the intervention. ERD = Event–Related Desynchronization; BCI = Brain–Computer Interface; iTBS = intermittent Theta–Burst Stimulation. T0 = baseline; T10 = post-training. *p < 0.05, **p < 0.01, ***p < 0.001.

**Figure 8:**
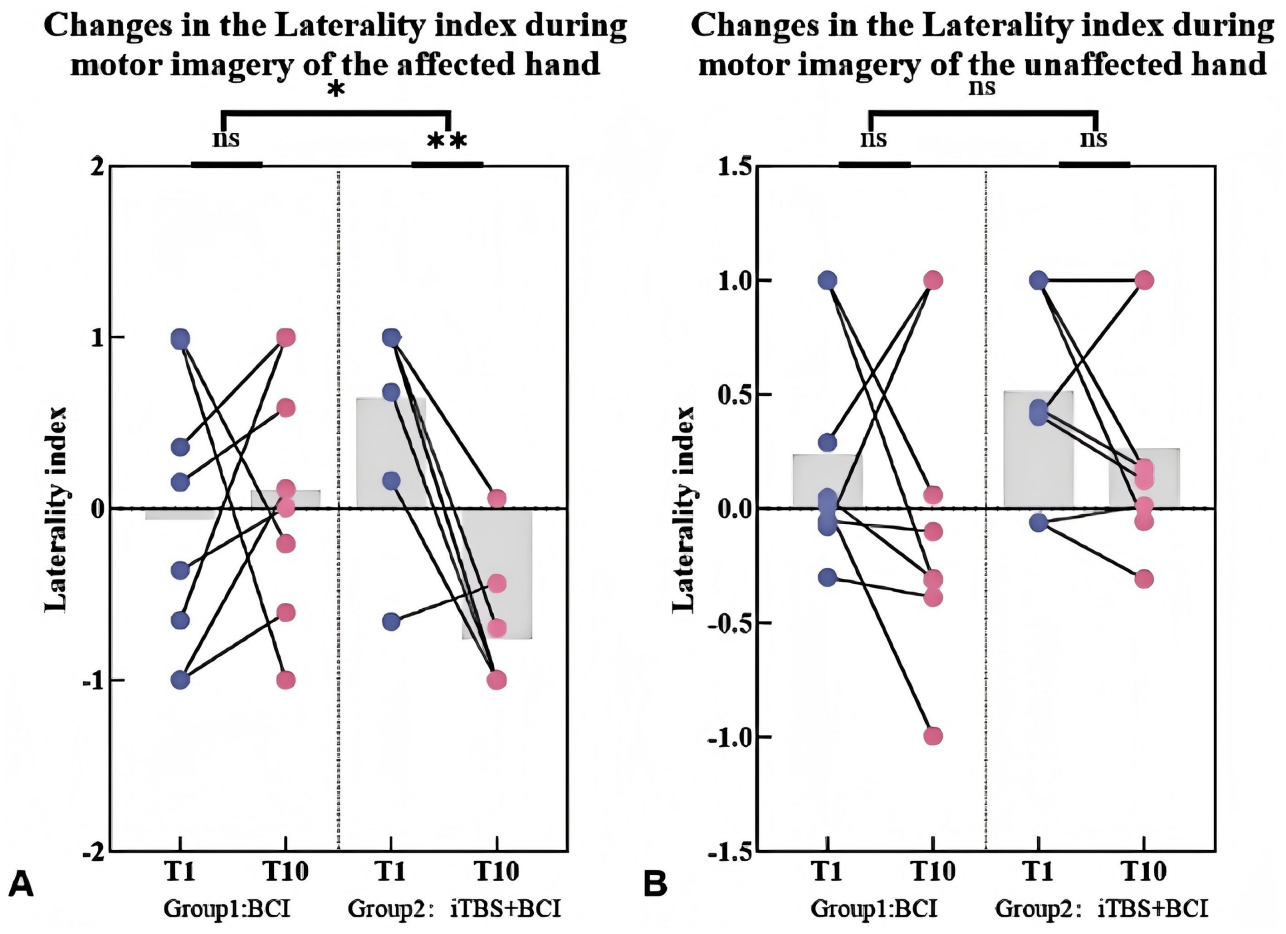
Changes in laterality index (LI) during motor imagery of affected and unaffected hands. Plots show the LI changes at T1 and T10 for two groups (Group 1: BCI; Group 2: iTBS + BCI) during motor imagery of the affected **(A)** and unaffected **(B)** hands. Connected dots represent individual participants. Abbreviations: BCI = Brain–Computer Interface; iTBS = intermittent theta-burst stimulation. T0 = baseline; T10 = post-training. *p < 0.05, **p < 0.01, ***p < 0.001, ns = not significant.

Analysis of ERD and LI in the sensorimotor cortices of both groups at baseline (T0) and post-training (T10) showed that during MI of the affected hand: C3-ERD Showed no significant change within Group 1 (*P₁* = 0.965), within Group 2 (*P₂* = 0.137), or between groups (*P₃* = 0.446). C4-ERD Showed no significant change within Group 1 (*P₁* = 0.514), but showed significant changes within Group 2 (*P₂* = 0.001) and between groups (*P₃* = 0.001). LI Showed no significant change within Group 1 (*P₁* = 0.688), but showed significant changes within Group 2 (*P₂* = 0.001) and between groups (*P₃* = 0.017). During MI of the unaffected hand, C3-ERD, C4-ERD, and LI showed no significant differences within or between groups for either measure (all *p* > 0.05) (Figures 7A–D, 8A,B and Table 3). This suggests that after the combined iTBS + BCI intervention, MI of the affected hand resulted in a deepening of C4-ERD and a shift of the LI towards the affected hemisphere. A deeper ERD reflects increased desynchronization of neural activity in the affected sensorimotor cortex, signifying enhanced cortical activation levels. A shift of the LI towards the affected hemisphere indicates an adjustment in the lateralization pattern of motor-related cortical activation, gradually remodeling towards a pattern more conducive to affected hand motor function (approaching the normal pattern). Together, these findings demonstrate that the combined intervention enhances cortical activity on the affected side, promotes neural plasticity, and facilitates a shift in the activation pattern of motor-related brain regions towards a state closer to normal, laying an electrophysiological foundation for the improvement of affected upper limb motor function.

**Table 3:** Changes in ERD and LI between T0 and T10.

Outcome measure	Group1			Group2			*P3*
	T0	T10	*P_1_*	T0	T10	*P_2_*	
Affected hand C3 ERD	0.0269 ± 0.5302	0.0130 ± 0.3746	0.965	−0.1180 ± 0.3341	0.1502 ± 0.3680	0.137	0.446
Affected hand C4 ERD	0.0764 ± 0.3641	0.1063 ± 0.3424	0.514	0.2101 ± 0.1182	−0.2449 ± 0.1696	0.001	0.001
Affected hand LI	−0.0642 ± 0.8139	0.1127 ± 0.7238	0.688	0.6483 ± 0.6050	−0.7585 ± 0.3916	0.001	0.017
Unaffected hand C3 ERD	−0.0508 ± 0.6077	−0.0282 ± 0.2991	0.942	−0.0833 ± 0.3543	−0.1747 ± 0.2682	0.521	0.747
Unaffected hand C4 ERD	0.1798 ± 0.6769	−0.0539 ± 0.2724	0.398	0.2241 ± 0.3673	−0.0774 ± 0.2935	0.163	0.819
Unaffected hand LI	0.2397 ± 0.4966	−0.0049 ± 0.6911	0.438	0.5167 ± 0.4466	0.2659 ± 0.4794	0.212	0.986

P₁ and P₂ indicate intragroup differences (T10 vs T0) for Group 1 and Group 2, respectively. P₃ represents intergroup differences in changes from T0 to T10. ERD = Event-Related Desynchronization; LI = Lateral Index; T0 = baseline; T10 = post-training.

## Discussion

This exploratory study is the first to preliminarily investigate the potential synergistic effects of applying iTBS to the affected M1 of stroke patients before BCI training in stroke patients. The results showed that the combined intervention was superior to BCI monotherapy in improving upper limb motor function and reducing limb muscle tone, with a clear time-dependent therapeutic effect. More importantly, the combined intervention enhanced BCI task accuracy and patient attention, and the magnitudes of these improvements showed a negative correlation. Neurophysiological evidence further revealed that the combined intervention enhanced the desynchronization (deepening of C4-ERD) in the affected sensorimotor cortex during MI of the affected hand and promoted a lateralization shift of motor-related brain region activation patterns towards the normal pattern.

Clinical benefit analysis revealed that the mean improvement in FMA-UE score in the combined group reached 24.67 points at week 4, exceeding the widely recognized minimal clinically important difference (MCID) of 5 points (41). The efficacy was significantly superior to previously reported single-modality treatments with BCI or iTBS, confirming a synergistic effect between the two interventions (5, 42–44). The lack of significant efficacy in week 2 may be attributed to the time required for the induction of neuroplasticity and the manifestation of sequential synergistic effects. This study found that the combined intervention significantly reduced limb muscle tone. The underlying mechanism may involve the modulation of the corticospinal descending inhibitory pathway by iTBS and the correction of abnormal motor patterns by BCI training. The absence of intergroup differences in MBI and emotional scores may be related to the lag of activities of daily living improvement behind motor function recovery, short intervention duration, small sample size, and the fact that the intervention target did not directly act on brain regions involved in emotional regulation.

Consistent with previous research on rTMS combined therapies, the current study found that iTBS significantly improved BCI training accuracy. The underlying mechanism likely involves multi-level neural modulation (25, 45). Firstly, TMS, by enhancing excitability in the M1, may optimize the generation and decoding efficiency of motor intention-related EEG signals, thereby improving the BCI system’s recognition accuracy of patient MI (46–48). Secondly, iTBS-induced enhancement of synaptic plasticity may promote the stability of functional connectivity within the sensorimotor network, making the neural feedback during BCI training more precise (49, 50). Additionally, improved attention may indirectly contribute to this process, as iTBS may remotely modulate the dorsolateral prefrontal cortex to enhance cognitive engagement (51). By directly enhancing excitability in motor-related brain regions and remotely modulating attention, iTBS provides a more efficient neural signal basis for BCI training. Future research should integrate functional magnetic resonance imaging (fMRI) to directly analyze the impact of iTBS on the dynamics of MI-related brain networks.

This study further found a significant negative correlation between the magnitude of BCI accuracy improvement and the reduction in Schulte Grid Test time, indicating a synergistic relationship between enhanced patient attention and improved BCI decoding efficiency. Patients with greater attention enhancement exhibited higher-quality neural signals during motor imagery and correspondingly higher BCI accuracy, thereby forming a virtuous cycle of “cognitive enhancement-motor improvement”. This finding complements the cognitive dimension of the “BCI illiteracy” explanation, demonstrating that post-stroke attention deficits are also a key factor limiting BCI efficacy. It also provides preliminary clinical evidence for the cross-brain-region remote modulatory effect of iTBS via the frontoparietal attention network.

This study found that sequential iTBS and BCI treatment significantly deepened the amplitude of ERD in the affected sensorimotor cortex (C4 region) during MI of the affected hand and drove the LI toward the affected hemisphere (the physiological activation direction). The possible mechanism underlying this neural remodeling may involve modulation of the affected M1 cortex by iTBS. Its theta-rhythmic burst stimulation induces Long-Term Potentiation (LTP) effects, transiently increasing cortical excitability and lowering the neural activation threshold for motor imagery. This allows patients to more effectively recruit resources from the affected motor network during subsequent BCI training (49). This “excitability pre-activation” effect synergizes with the closed-loop feedback of BCI, collectively promoting a functional shift of motor control back towards the affected hemisphere’s dominant pathway. Notably, previous studies observed that MI of the unaffected hand increased ERD values in the affected hemisphere and induced an LI shift towards the unaffected (normal) hemisphere (40). While this shares the task-specificity pattern observed here, it reflects a different direction of neural remodeling. MI of the unaffected hand relies on the physiological activation pattern dominated by the unaffected hemisphere, with increased ERD reflecting the normal functional maintenance of the unaffected network. Conversely, MI of the affected hand must overcome post-stroke compensatory overactivation of the unaffected hemisphere; an LI shift towards the affected hemisphere signifies the reconstruction of autonomous control capabilities within the affected network. This study focused on the neural response during affected hand MI, directly addressing the core challenge in stroke rehabilitation: utilizing targeted excitability modulation by iTBS and task-specific BCI training to transform from “abnormal unaffected hemisphere compensation” to “affected hemisphere dominant control.” This provides a new pathway to address “BCI illiteracy” (stemming from insufficient *μ*-ERD signals). Compared to the limitations of tDCS-BCI approaches where accuracy improvements failed to translate into functional gains, the efficient induction of synaptic plasticity by iTBS may be the key advantage enabling its synergy with BCI to achieve neural remodeling and functional translation (52, 53).

## Limitations and future directions

This study has several limitations. First, the absence of a sham iTBS control group prevents us from definitively distinguishing the specific neuroplastic effects of iTBS from non-specific placebo or attention-related effects, so the specific therapeutic contribution of iTBS remains to be confirmed. Secondly, the small sample size results in limited statistical power and cannot completely eliminate the risks of Type I and Type II errors, thus all statistical results should be interpreted with caution. Additionally, the enrollment of both ischemic and hemorrhagic stroke patients without stratified analysis may introduce clinical heterogeneity, which could interfere with the interpretation of neurophysiological outcomes. Thirdly, potential confounders such as medication use and spontaneous recovery were not strictly controlled, and the lack of long-term follow-up limits assessment of treatment effect sustainability. Future research should validate the long-term benefits of the combined intervention by expanding the sample size and setting multiple assessment time points.

Future research should address these limitations by conducting large-scale, sham-controlled randomized trials with subgroup analyses, integrating long-term follow-up to validate the sustainability of benefits and clarify the generalizability of the combined intervention strategy.

## Conclusion

This exploratory study provides preliminary evidence that iTBS combined with BCI therapy may improve upper limb motor function and reduce limb muscle tone in stroke patients, with efficacy unaffected by baseline motor function level. The mechanism of action is related to the synergistic effect between iTBS-induced cortical plasticity enhancement and task-specific neural remodeling driven by BCI training. These results offer a novel combined intervention strategy for stroke rehabilitation with significant clinical translational value.

## Data Availability

The original contributions presented in the study are included in the article/supplementary material, further inquiries can be directed to the corresponding author/s.
